# Cutaneous malignant melanoma “recurred as” or “in coexistence” with meningioma?

**DOI:** 10.4103/2152-7806.70853

**Published:** 2010-10-06

**Authors:** Nunung Nur Rahmah, Tetsuyoshi Horiuchi, Jun Nakayama, Junpei Nitta, Kazuhiro Hongo

**Affiliations:** Department of Neurosurgery Shinshu University School of Medicine, Matsumoto, Japan; 1 Department of Pathology, Shinshu University School of Medicine, Matsumoto, Japan

**Keywords:** Malignant melanoma, meningioma, neural crest, recurrence, coexistence-losomarginal artery

## Abstract

**Background:**

The authors report a rare case of a patient with previously treated cutaneous malignant melanoma that recurred 1 year later as an intracranial meningioma.

**Case Description:**

A 20-year-old woman presented with exophthalmos, diplopia and a mass in the left supraorbital area. Imaging study revealed an enhanced intracranial extradural mass with bone destruction. The patient had a history of cutaneous malignant melanoma surgery on the same location 1 year before. The patient underwent left frontotemporal craniotomy for total resection of the mass. Histological study revealed the intracranial mass to be an atypical meningioma.

**Conclusion:**

To our knowledge, this is a rare report of a patient with this tumor occurrence. This case serves to remind neurosurgeons of the potential existence of benign and/ or malignant tumors of neural crest origin.

## INTRODUCTION

Cutaneous melanoma is an increasingly prevalent disease. It is currently the third most common cause of cerebral metastases after lung and breast cancers.[1] Intracranial metastases of melanomas have been well documented; however, intracranial meningioma as a recurrence of cutaneous malignant melanoma is seldom found. We report a rare case of a frontal meningioma discovered in a patient with a previously removed supraorbital cutaneous malignant melanoma.

## CASE REPORT

**Figure 1:**
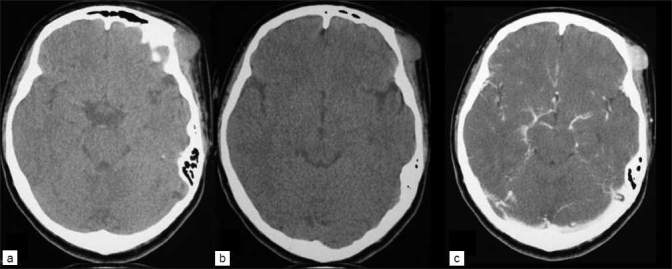
CT scan showing (a, b) precontrast images of extracranial mass of cutaneous malignant melanoma of the left supraorbital area with no bone involvement and (c) strongly enhanced melanoma

**Figure 2:**
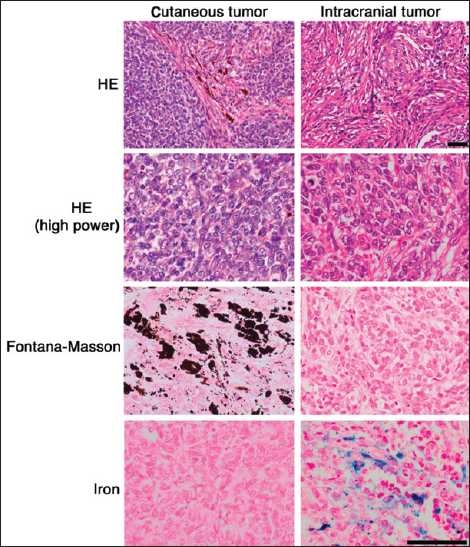
Comparison of histopathology between the cutaneous tumor and intracranial tumor. Images showing HE, Fontana-Masson, and iron stainings; scale bar = 50 mm

**Figure 3:**
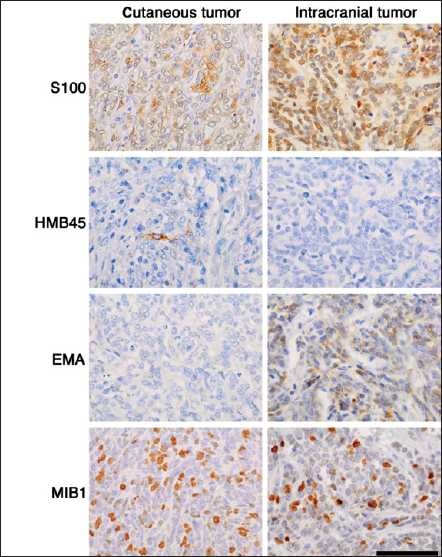
Comparison of histopathology between the cutaneous tumor and intracranial tumor. Images showing immunohistochemistry for S100 protein, HMB45, EMA and MIB1; scale bar = 50 mm

A 20-year-old Japanese woman had a history of cutaneous malignant melanoma on the left supraorbital area. Computed tomography (CT) scan showed no bone and intracranial involvement [Figure 1]. Macroscopically, pigmentation was not obvious on the skin covering the cutaneous tumor. Simple resection was carried out to establish the histological diagnosis. The epidermis was left intact for cosmetic reason. Histopathological examination revealed that the tumor was composed of sheet-like proliferation of small round cells which had pleomorphic nuclei and prominent nucleoli [Figure 2]. Occasionally, melanin pigment, which was confirmed by Fontana-Masson staining, was found in the tumor cells. Mitotic figures were frequently found (5/10 HPF), but necrotic foci were not detected. Immunohistochemistry revealed that these tumor cells were moderately positive for S100 protein and focally positive for HMB45, but negative for Melan-A and epithelial membrane antigen (EMA) [Figure 3]. Details of these cell-specific markers are shown in Table 1. MIB1 index was 25.9%. These findings were characteristic of malignant melanoma.

**Table 1:** Cell-specific markers examined in this study

Markers	Description	Tumors
S-100	Calcium binding protein	Melanoma, schwannoma, astrocytoma, oligodendroglioma, ependymoma, granular cell tumor, meningioma
HMB-45	Melanocytic protein	Melanoma, angiomyolipoma
Melan-A	Melanocytic protein	Melanoma, angiomyolipoma, adrenocortical carcinoma
EMA	Epithelial protein	Carcinoma, meningioma, ependymoma

Postoperative gallium scintigraphy scanning did not reveal any recurrence or metastasis. No chemotherapy or radiotherapy was performed, because informed consent could not be obtained. One year later, she was referred to our service because of exophthalmic left eye and diplopia. She had no family history of melanoma or other malignancies. Visual acuities were normal, external eye movement of the left eye was slightly reduced and other neurological findings were normal.

**Figure 4:**
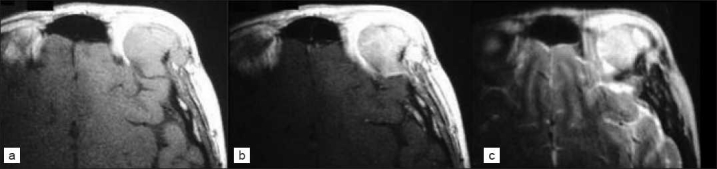
CT scan (a, b) postcontrast images showing enhanced intracranial mass of the supraorbital area with bone involvement and (c) bone window image showing bone destruction by the mass

**Figure 5:**
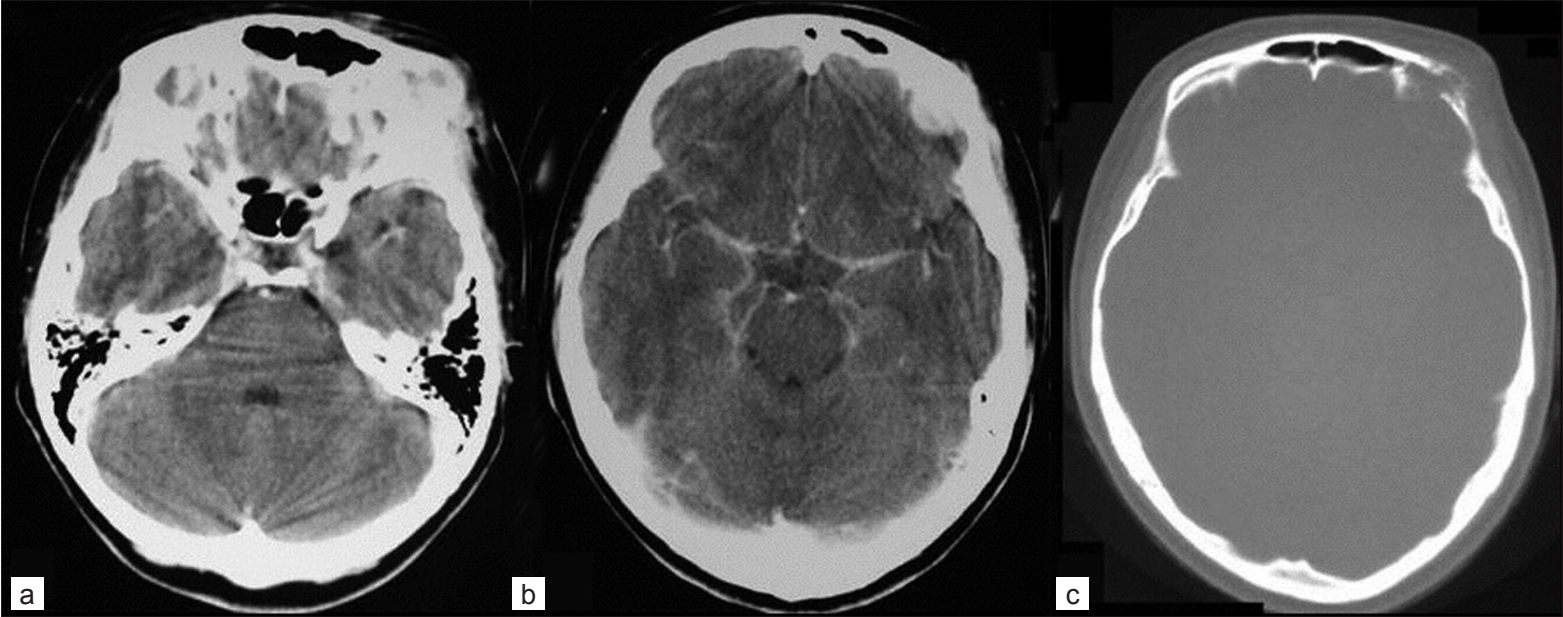
MRI study showing a) an isointense 3.5 × 2.5 cm mass on axial T1-weighted image, b) T1 with contrast, c) hyperintense mass with marked border to its surrounding and appearance of bone destruction on T2 weighted image

CT scan showed an enhanced mass on the left supraorbital area with bone involvement [Figure 4]. Magnetic resonance imaging (MRI) clearly showed a 3.5 cm × 2.5 cm enhanced mass with marked border to its surrounding brain structures. The mass also caused bone destruction and bulged to subcutaneous tissue [Figure 5]. Thus, our preoperative diagnosis was intracranial invasion of melanoma. No other metastatic lesion was found on examination.

Left frontotemporal craniotomy was performed. During the surgery, the tumor was found to be black, soft, easy to bleed and was tightly adhered to the dura. Total resection of the tumor was achieved. No surgery-related complication was found postoperatively. No adjuvant therapy was given to the patient postoperatively. Histology of the intracranial tumor indicated the close proliferation of short spindle tumor cells focally, showing a vague wheel-like formation [Figure 2]. Mitotic figures were frequently found (4/10 HPF), but tumor necrosis was absent. Iron staining demonstrated focal hemosiderin deposition in the tumor, suggestive of hemorrhage. Immunohistochemistry of the tumor cells revealed that they were strongly positive for S100 protein and moderately positive for EMA, but negative for HMB45 and Melan-A [Figure 3]. MIB1 index was 24.5%. These results established that the intracranial tumor was atypical meningioma.

Postoperative condition was uneventful with normal neurological findings. External eye movement was improved. One year later, the patient was admitted to hospital because of liver metastases of melanoma. Histology showed characteristics of malignant melanoma. She died in the same year.

## DISCUSSION

Melanoma has a propensity for multiorgan involvement, and central nervous system complications are frequent.[1] The most common site of recurrence is brain parenchyma;[2,1] however, there are only a few reports regarding its metastases to skull, meninges, or spine.[3–6] The patient had a removal history of cutaneous. tumor a year earlier. Histologically, the tumor cells had characteristic features consistent with malignant melanoma. Fontana-Masson staining revealed that they had melanin pigments in the cytoplasm, and immunohistochemistry demonstrated that S100 and HMB45 were positive. It was noted that Melan-A was negative in this case, however, the absence of Melan-A could not exclude the possibility for malignant melanoma because Jungbluth *et al*. reported that approximately 20% of melanomas were negative for Melan-A.[7]

Preoperatively, the mass was presumed to be an intracranial recurrence of melanoma since the imaging findings of intracranial melanoma can vary from hypointensity to hyperintensity to its surrounding brain structures, and slightly to homogeneously enhanced mass.[8–10] Even though it is rare, it has been mentioned in the literature that imaging appearance of bony involvement in melanoma can be either as a hypointense or hyperintense mass on T_1_-weighted image, or as a compressed fracture lesion,[11,2] therefore raising the possibility of the hyperintense mass and its bony involvement to be a melanoma. One theory explaining the tendency of bony involvement in melanoma is that tumor associated macrophages in melanoma are capable of osteoclast differentiation by receptor activator for nuclear factor kB ligand dependent mechanism, thereby causing tumor osteolysis.[12]

Histopathological study of this case revealed an atypical meningioma, which was completely beyond our expectation. Tumor to tumor metastases have been documented;[11,13,14] however, there are only five cases reported concerning melanoma and all these reports emphasized on the existence of melanoma cells in the previously existed intracranial host tumor of different origin, which is different from our case. There were no melanoma cells found in the meningioma even though macroscopic appearance was black, which can mean that either the melanoma invaded intracranially and completely changed into meningioma or the meningioma had already existed when melanoma was diagnosed, but it was too small to be discovered from imaging studies. We also performed iron staining to exclude the possibility of recurrence of melanoma, and the result was positive which meant that the black color found during surgery was an old hemorrhage [Figure 2]. There are some reports of occurrence of meningioma as a second tumor due to radiotherapeutic effects;[14] however, our patient did not undergo any radio- or chemotherapy after simple resection of melanoma, thereby excluding radiotherapeutic effect as a risk factor for occurrence of meningioma.

Of the first possibility mentioned above, tumor to tumor metastasis, mechanisms of its changes are not well understood. Baruch mentioned the roles of chemokines and their receptors in organ selectivity in metastasis.[15] In this case, the chemokines and their receptors might have selected the dura as their metastatic location, but then the chemokines have undergone certain mechanism involving meningioma receptor causing them to develop into a different type of tumor, which is meningioma in this case. Further study is needed to obtain a complete understanding of the pathways involved.

Another possibility is the coexistence of melanoma and meningioma. Warwar *et al*. reported a 64-year-old woman who was referred for evaluation of diabetic retinopathy and was found with right superolateral periorbital neurofibroma, a right sphenoid wing meningioma, and a left choroidal juxtapapillary malignant melanoma. The patient then underwent excision for the periorbital mass and the meningioma. Eye enucleation was performed to the choroidal tumor. No recurrence was found during 1-year follow-up.[16] The peripheral nervous system, leptomeninges and melanocytes originate from neural crest cells; therefore, it is possible that later in life they can form and develop into such tumors mentioned above at the same time. This disorder is called neurocristopathies.[16] Perhaps it can be applied to our patient. It certainly was not cristopathies because before the first surgery there was no obvious neurofibroma and meningioma found on examination, but the chance of such a small meningioma undetected through imaging studies does exist. In summary, despite its rare occurrence, melanomas which recur as or in coexistence with another form of tumor deserve high precaution since low survival rate of melanoma with intracranial involvement has been well documented.[1,2] This case serves to remind neurosurgeons to be aware of potential existence of other benign and/or malignant tumors of neural crest origin.
